# Association Between Serum IL-6 Levels and Disease Severity Among COVID-19 Patients Hospitalized at a Tertiary Care Hospital in Bangladesh

**DOI:** 10.7759/cureus.102631

**Published:** 2026-01-30

**Authors:** Sadia Amin, Nisat Zabin, Noureen Amin, Sheikh Nazmul Islam, Taiyeb I Zahangir, Sumona Islam, Mahnaz Islam, Amit Bari

**Affiliations:** 1 Department of Respiratory Medicine, Bangabandhu Sheikh Mujib Medical University, Dhaka, BGD; 2 Department of Nephrology, Bangabandhu Sheikh Mujib Medical University, Dhaka, BGD; 3 Department of Psychiatry, National Institute of Mental Health and Hospital, Dhaka, BGD; 4 Department of Gastroenterology, Bangabandhu Sheikh Mujib Medical University, Dhaka, BGD; 5 Department of Nephrology, Dhaka Medical College Hospital, Dhaka, BGD; 6 Department of Nephrology, Kidney Foundation Hospital and Research Institute, Dhaka, BGD

**Keywords:** coronavirus disease 2019, covid-19, covid-19 severity, interleukin-6, sars-cov-2

## Abstract

Introduction

COVID-19 severity varies widely among patients, and early identification of those at risk for severe disease is essential. IL-6, a key inflammatory cytokine, has been implicated in the pathogenesis of severe COVID-19. This study aimed to assess the association between serum IL-6 levels and disease severity among hospitalized COVID-19 patients at Bangabandhu Sheikh Mujib Medical University (BSMMU).

Methodology

A cross-sectional study was conducted among 90 RT-PCR-confirmed COVID-19 patients admitted to BSMMU. Patients were categorized into non-severe, severe, and critical groups based on WHO guidelines. Serum IL-6 levels and routine laboratory parameters were measured. Statistical analyses included chi-square tests, correlation analysis, logistic regression, and ROC curve evaluation to determine predictors of severity.

Results

Higher IL-6 levels were significantly associated with increasing disease severity. Severe and critical patients were older and more likely to have diabetes or hypertension. IL-6 showed positive correlations with neutrophil count, CRP, ferritin, D-dimer, and lactate dehydrogenase (LDH), and a negative correlation with lymphocyte count. Logistic regression identified IL-6 >14 pg/mL as an independent predictor of severe or critical disease. Receiver operating characteristic (ROC) analysis demonstrated excellent diagnostic performance of IL-6 in predicting severe outcomes.

Conclusion

Elevated IL-6 levels correlate strongly with COVID-19 severity and independently predict severe or critical illness. IL-6 may serve as an effective biomarker for early risk stratification, enabling timely intervention and improved patient management. Further studies are recommended to validate these findings and explore IL-6-targeted therapies.

## Introduction

COVID-19 is an infectious illness caused by SARS-CoV-2, which was first identified in Wuhan, China, in late 2019 and subsequently spread worldwide, resulting in a major public health emergency [1,2]. The WHO officially characterized COVID-19 as a pandemic on March 11, 2020, following its rapid transcontinental spread, which placed substantial strain on healthcare systems and resulted in considerable morbidity and mortality worldwide [3,4]. Bangladesh documented its first laboratory-confirmed case of COVID-19 on March 8, 2020. Since then, the virus has continued to spread throughout the country. A notable second wave began in March 2021, marked by a sharp increase in infections across various regions [5,6].

SARS-CoV-2 is an enveloped, single-stranded RNA virus that enters human cells through the ACE2 receptor, which is widely expressed not only in the respiratory tract but also in the kidneys, heart, gastrointestinal system, and other organs [7,8]. This widespread expression accounts for the virus's ability to cause multi-organ involvement. While many individuals experience mild or moderate symptoms, a substantial proportion develop severe complications such as pneumonia, acute respiratory distress syndrome (ARDS), and multi-organ failure [7,9,10].

A major factor contributing to the severity of COVID-19 is the dysregulated immune response, particularly the development of a "cytokine storm." This phenomenon is characterized by the excessive release of pro-inflammatory cytokines, including IL-6, which plays a central role in mediating inflammation and immune responses [11-14]. Elevated IL-6 levels have been associated with disease progression, respiratory failure, need for intensive care, and increased mortality in COVID-19 patients [15,16,17].

Numerous studies have indicated that IL-6 may serve as a valuable biomarker for predicting disease severity and clinical outcomes in hospitalized patients [18]. Early identification of patients with elevated IL-6 levels could allow timely intervention and improved management strategies. Given the high burden of COVID-19 in Bangladesh and the pressing need for effective risk stratification tools, this study aims to explore the association between serum IL-6 levels and disease severity among hospitalized COVID-19 patients at Bangabandhu Sheikh Mujib Medical University (BSMMU).

## Materials and methods

Study design

A cross-sectional study was conducted to assess the association between serum IL-6 levels and disease severity among hospitalized COVID-19 patients. Patients were selected based on predefined inclusion and exclusion criteria. Data were collected from individuals admitted to the COVID-19 Unit of Bangabandhu Sheikh Mujib Medical University (BSMMU). No follow-up was conducted after the initial evaluation.

Patient recruitment

Patients were recruited from the COVID-19 Unit of BSMMU, Dhaka, Bangladesh, over a six-month period from June to December 2021. The study population comprised hospitalized patients with laboratory-confirmed COVID-19 diagnosed by RT-PCR. Eligible participants were adults aged 18 years or older of either sex, with a duration of illness ranging from 7 to 14 days, who provided written informed consent. Patients were excluded if they were unwilling to participate, had evidence of bacterial infection (procalcitonin level >0.5 ng/mL), had received IL-6 antagonist therapy (such as tocilizumab), had a history of rheumatological diseases including rheumatoid arthritis or systemic lupus erythematosus, or were pregnant.

Data collection procedure

Eligible participants were informed about the aims of the study, study procedures, and their rights, including the option to withdraw at any stage without prejudice. After obtaining written informed consent, demographic data (age, sex, occupation, educational status, and smoking history), comorbid conditions (such as diabetes mellitus, hypertension, chronic lung disease, chronic kidney disease, and ischemic heart disease), and presenting clinical features (including fever, cough, dyspnea, sore throat, and fatigue) were documented.

A comprehensive physical examination was conducted. Peripheral oxygen saturation was measured using a pulse oximeter. Disease severity was categorized in accordance with the WHO COVID-19 Clinical Management: Living Guidance [3].

Five milliliters of venous blood was collected aseptically from each participant to perform tests including complete blood count (CBC) with ESR, D-dimer, IL-6, CRP, lactate dehydrogenase (LDH), and ferritin. Samples were sent to the Microbiology Laboratory of BSMMU. IL-6 levels were measured within 48 hours of admission. Based on WHO criteria, patients were categorized into three groups: non-severe, severe, and critical [3]. Convenience sampling was used, and 30 patients were recruited in each group. All collected data were entered into a pre-designed data collection sheet.

Data analysis

Data were checked for completeness and consistency, and incomplete records were excluded. Quantitative and qualitative variables were analyzed using IBM SPSS version 23. Continuous variables were expressed as mean ± SD, while categorical variables were summarized as frequencies and percentages. Group comparisons were performed using unpaired t-tests for continuous variables and chi-square tests for categorical variables. Pearson’s correlation coefficient was used to assess the relationship between IL-6 levels and other variables. Binary logistic regression was applied to identify predictors of disease severity. ROC curve analysis was performed to evaluate the predictive value of IL-6 for severe and critical COVID-19. A p-value <0.05 was considered statistically significant.

## Results

This cross-sectional study was conducted in the COVID unit of BSMMU from June 2021 to December 2021. A total of 90 patients were included in this study based on the inclusion and exclusion criteria. The main objective was to determine the relationship between IL-6 and disease severity among COVID-19 patients hospitalized at BSMMU.

The demographic characteristics and co-morbidities of the study population are summarized in Table 1.

**Table 1:** Demographic characteristics and comorbidities. IHD: Ischemic heart disease; CKD: Chronic kidney disease.

Variables	Total (n=90)	Non-severe (n=30)	Severe (n=30)	Critical (n=30)	P-value
Age (mean ± SD)	56.9 ± 14.4	48.5 ± 12.5	56.4 ± 13.8	61.4 ± 15.6	0.04
Gender: Male	54 (60%)	18 (60%)	17 (56.7%)	19 (63.3%)	0.97
Gender: Female	36 (40%)	12 (40%)	13 (43.3%)	11 (36.7%)	
Smoking status: Smoker	42 (46.7%)	13 (43.3%)	14 (46.7%)	15 (50%)	0.7
Smoking status: Non-smoker	48 (53.3%)	17 (56.7%)	16 (53.3%)	15 (50%)	
Comorbidities: Hypertension	54 (60%)	10 (33.3%)	14 (46.7%)	30 (100%)	<0.001
Comorbidities: Diabetes	71 (78.9%)	17 (56.6%)	24 (80%)	30 (100%)	0.002
Comorbidities: IHD	46 (51.1%)	6 (20%)	10 (33.3%)	30 (100%)	<0.001
Comorbidities: CKD	16 (17.8%)	0	4 (13.3%)	12 (40%)	<0.001
Comorbidities: Preexisting lung disease	11 (12.2%)	3 (10%)	6 (20%)	2 (6.7%)	0.26

ANOVA showed that there was a statistically significant difference in age distribution and the prevalence of most comorbidities among the three groups (p-values mentioned in the table), with patients in the severe and critical groups being older and having higher comorbidity.

In Table 2, ANOVA showed that there was a statistically significant difference in neutrophil count, lymphocyte count, CRP, ferritin, D-dimer, LDH, and IL-6 among the three groups (p-values mentioned in the table), with patients in the severe and critical groups having higher values (except lymphocyte count, which showed the opposite trend).

**Table 2:** Laboratory parameters according to COVID-19 severity. Hb: Hemoglobin; LDH: Lactate dehydrogenase.

Parameter	All patients (N=90)	Non-severe (n=30)	Severe (n=30)	Critical (n=30)	P-value
Hb (g/dL)	11.9 ± 1.7	12.1 ± 1.3	12.1 ± 2.2	11.4 ± 1.1	0.212
ESR (mm/hr)	54.5 ± 36.2	47.8 ± 25.8	55.0 ± 35.7	59.5 ± 44.1	0.485
WBC count (×10³/µL)	12.1 ± 9.3	11.1 ± 13.5	11.4 ± 9.4	13.6 ± 2.1	0.548
Neutrophil count (%)	81.8 ± 11.7	75.4 ± 12.2	81.4 ± 11.8	87.8 ± 7.8	<0.001
Lymphocyte count (%)	13.3 ± 10.4	19.0 ± 11.3	14.7 ± 10.8	6.8 ± 4.1	<0.001
Platelet (×10³/µL)	259 ± 84	248 ± 77	274 ± 107	252 ± 56	0.412
Mean IL-6 (pg/mL)	51.0 ± 52.8	11.4 ± 43.5	37.1 ± 41.8	101.1 ± 26.7	<0.001
CRP (mg/L)	82.5 ± 51.5	46.1 ± 39.1	88.3 ± 61.0	107.7 ± 27.6	<0.001
Ferritin (µg/L)	1236.1 ± 1877.6	552 ± 534	1155 ± 1198	1919 ± 2833	0.022
D-dimer (µg/mL)	2.27 ± 2.85	0.45 ± 0.44	1.29 ± 2.0	4.9 ± 2.9	<0.001
LDH (IU/L)	399 ± 352.2	292.6 ± 1	313.4 ± 17	586.3 ± 525	0.001

In Table 3, the Chi-square test showed that there was a statistically significant difference in IL-6 elevation among the three groups (p < 0.0001), with the severe and critical groups having a higher percentage of patients with elevated IL-6.

**Table 3:** Association between IL-6 and COVID-19 severity (N = 90).

Severity of COVID-19	Normal IL-6 (n=30)	Elevated IL-6 (n=60)	P-value
Non-severe	26 (86.7%)	4 (6.7%)	<0.001
Severe	4 (13.3%)	26 (43.3%)	
Critical	0 (0.0%)	30 (50.0%)	

In Table 4, Pearson’s correlation showed a significant positive correlation between IL-6 levels and age, WBC count, neutrophil count, CRP, ferritin, D-dimer, and LDH, while showing a significant negative correlation between IL-6 levels and lymphocyte count (p-values mentioned in the table).

**Table 4:** Correlation between IL-6 levels and different parameters. Hb: Hemoglobin; LDH: Lactate dehydrogenase.

Variable	Pearson correlation coefficient	P-value
Age (years)	0.256	0.015
Hb (g/dL)	-0.135	0.205
ESR (mm/hr)	-0.057	0.594
WBC count (thousand/cmm)	0.211	0.046
Neutrophil count (%)	0.45	<0.001
Lymphocyte count (%)	-0.462	<0.001
Platelet count (thousand/cmm)	-0.135	0.203
CRP (mg/L)	0.397	<0.001
Ferritin (µg/L)	0.248	0.018
D-dimer (µg/L)	0.51	<0.001
LDH (IU/L)	0.33	0.002

**Figure 1:**
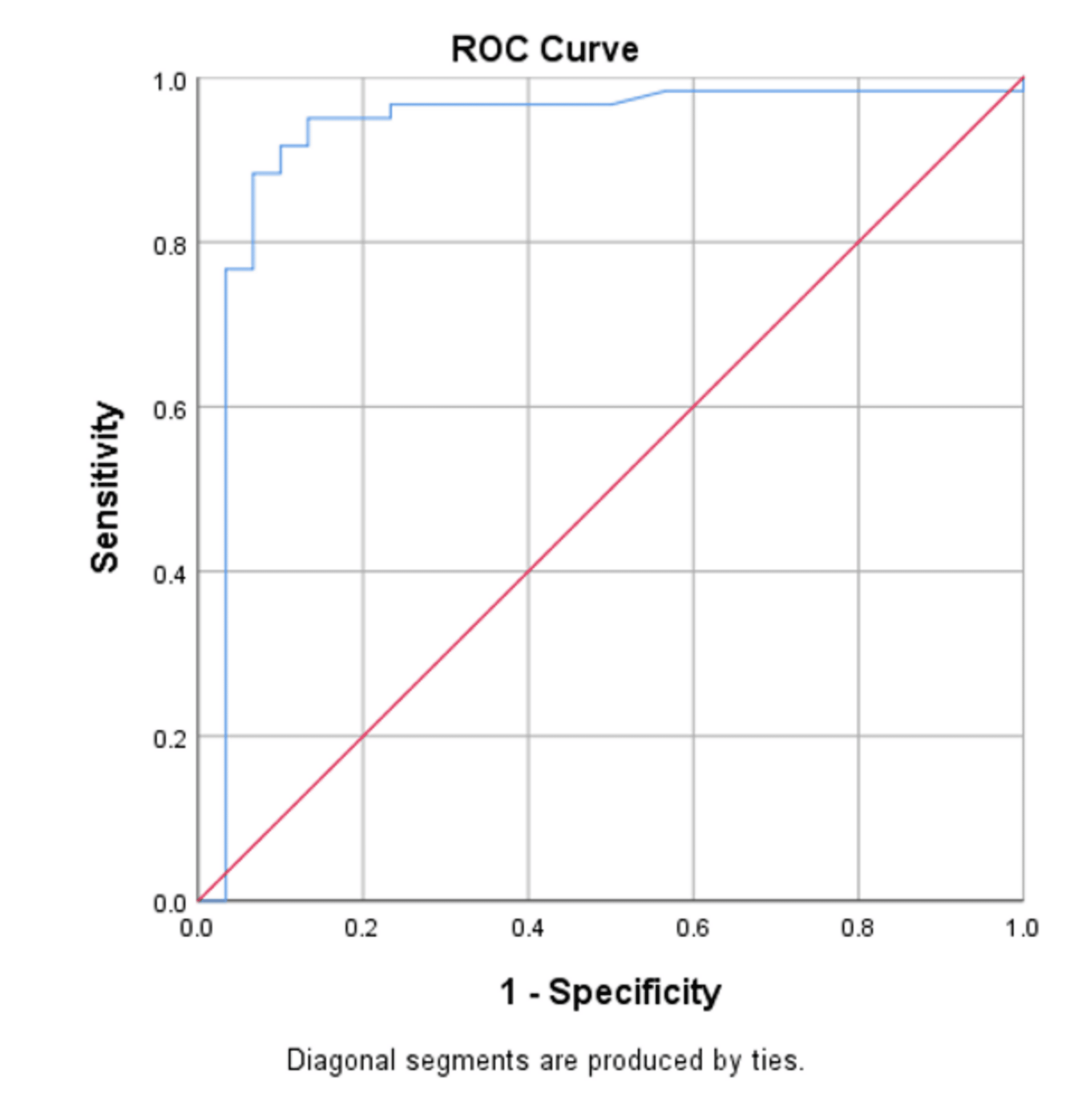
ROC curve of IL-6 level > 14.0 pg/mL for predicting the development of severe and critical COVID-19 in hospitalized patients (area under the curve = 0.927).

Table 5 shows the evaluation of IL-6 level >14 pg/mL for predicting the development of hypoxemia, with a sensitivity of 83.3% and specificity of 93.3%. The positive and negative predictive values were 96.2% and 73.6%, respectively. The accuracy of an IL-6 level >14 pg/mL for predicting the development of severe and critical cases in hospitalized patients was 86.7%. The area under the curve was 0.927 (95% CI: 0.846-1.000). ROC analysis demonstrated excellent diagnostic performance of IL-6 in predicting severe and critical COVID-19 (AUC = 0.927) (Figure 1).

**Table 5:** Sensitivity, specificity, PPV, NPV, and accuracy of IL-6 level >14 pg/mL for predicting the development of severe and critical COVID-19 in hospitalized patients. PPV: Positive predictive value; NPV: Negative predictive value.

Measure	Estimate (95% CI)
Sensitivity	83.3% (95% CI: 71.4%-91.7%)
Specificity	93.3% (95% CI: 77.9%-99.2%)
Positive predictive value	96.2% (95% CI: 86.7%-98.9%)
Negative predictive value	73.6% (95% CI: 61.2%-83.2%)
Accuracy	86.7% (95% CI: 77.8%-92.9%)
Area under the curve (AUC)	0.927 (95% CI: 0.846-1.000)

To assess the association between variables and to control for confounders, logistic regression analysis was performed. A p-value <0.05 was considered statistically significant.

Univariate logistic regression analysis showed that diabetes mellitus (DM), Hypertension (HTN), and IL-6 >14 were independent risk factors for COVID-19 severity. Multivariate logistic regression analysis (Table 6) showed that IL-6 >14 remained a risk factor after controlling for DM and HTN (OR: 20.197 for severe or critical disease when IL-6 >14, p=0.001).

**Table 6:** Regression analysis for COVID-19 severity. HTN: Hypertension.

Variables	OR	95% CI (Lower)	95% CI (Upper)	p-value
HTN	3.681	0.585	23.14	0.015
Diabetes mellitus	4.825	2.109	16.235	0.022
Ischemic heart disease	0.86	0.126	5.864	0.878
IL-6 > 14 pg/mL	20.197	6.238	68.261	0.001
Age > 50 years	1.006	0.95	1.065	0.846

## Discussion

This cross-sectional observational study evaluated the association between serum IL-6 levels and disease severity among hospitalized COVID-19 patients at BSMMU. The study included 90 RT-PCR-confirmed COVID-19 patients categorized into non-severe, severe, and critical groups. Our findings strongly suggest that elevated IL-6 levels correlate with increasing disease severity, reflecting the critical role of IL-6 in the pathogenesis of COVID-19.

In this study, most patients were in the fifth and sixth decades of life, with a mean age significantly higher in the severe and critical groups compared to the non-severe group. This is consistent with previous studies, including Aykal G et al., where older age was strongly associated with worse clinical outcomes. Age-related decline in immunity, increased prevalence of comorbidities, and chronic inflammation in elderly patients may collectively contribute to increased susceptibility [12]. These observations align with Zhou Y et al., who also found that hypertension, diabetes, CKD, and cardiac disease, conditions more common in older individuals, were associated with severe disease and increased mortality [1].

Male patients constituted 60% of our study population. Although men were more frequently affected, gender did not demonstrate a statistically significant relationship with COVID-19 severity. This finding corresponds with Liu T et al., indicating that while men are commonly infected, sex alone is not a major determinant of severity [19].

Smoking status showed an interesting trend. Although non-smokers were slightly more prevalent in the sample, smokers progressed to severe or critical illness more frequently. Previous studies have reported conflicting conclusions. Toussie D et al. suggested smoking worsens COVID-19 progression, whereas Korzeniowska A et al. hypothesised a potential protective effect due to nicotine’s interaction with ACE2 receptors [20,21]. Brake SJ et al., however, described upregulation of ACE2 receptors among smokers, increasing susceptibility [22]. Our findings support the latter, suggesting smokers may indeed be more vulnerable to severe outcomes.

Comorbidities played a major role in disease severity. Diabetes and hypertension were significantly more common in the severe and critical groups. This aligns with Honardoost M et al., who demonstrated that these comorbidities worsen COVID-19 outcomes [23]. Mechanistically, ACE2 expression in pancreatic β-cells may worsen glucose dysregulation during SARS-CoV-2 infection, and the imbalance of the renin-angiotensin system contributes to inflammation, oxidative stress, and upregulation of IL-6 [24].

The most important finding of our study was the strong association between IL-6 levels and disease severity. IL-6 levels were significantly elevated in severe and critical patients and showed positive correlations with neutrophilia, CRP, ferritin, D-dimer, LDH, and age, markers known to reflect inflammation and multi-organ involvement. Lymphocyte count showed a negative correlation, consistent with cytokine-mediated lymphocyte exhaustion. These results support earlier reports by Coperchini F et al., who emphasized IL-6 as a central mediator of cytokine storm and poor outcomes [25].

Our logistic regression analysis confirmed IL-6 >14 pg/mL as an independent predictor of severe or critical disease, even after adjusting for diabetes and hypertension, with an odds ratio of 20.2. Moreover, ROC analysis demonstrated high diagnostic accuracy, with an AUC of 0.927, highlighting IL-6 as a reliable biomarker for early risk stratification.

In summary, the findings of this study indicate a strong association between elevated IL-6 levels and increased disease severity among hospitalized patients with COVID-19. Measurement of IL-6 may therefore be useful for the early identification of high-risk individuals, facilitating prompt clinical intervention and optimized patient management.

This study has several limitations. The sample size was relatively small and the study was conducted at a single center, which may limit the generalizability of the findings. Additionally, the cross-sectional design prevents assessment of causal relationships or longitudinal changes in IL-6 levels over the course of illness. Certain confounding factors, such as variations in treatment and unmeasured comorbidities, could have influenced the results. Despite these limitations, the study provides valuable insights into the association between IL-6 levels and COVID-19 severity.

## Conclusions

This study shows that higher serum IL-6 levels are strongly associated with increased COVID-19 severity among hospitalized patients at BSMMU. Severe and critical cases were more common in older patients and those with diabetes or hypertension. IL-6 also correlated positively with inflammatory markers such as CRP, ferritin, D-dimer, and LDH, and negatively with lymphocyte count, reflecting its central role in the inflammatory response.

An IL-6 level >14 pg/mL was identified as an independent predictor of severe or critical disease, with excellent diagnostic accuracy. These findings highlight IL-6 as a useful biomarker for early risk assessment, helping clinicians identify high-risk patients who may need closer monitoring and timely intervention. Further studies are recommended to confirm these results and explore the benefits of IL-6-targeted therapies.
